# Timing of Fracture Fixation in Ankle Fracture-Dislocations

**DOI:** 10.1177/19386400241273105

**Published:** 2024-10-05

**Authors:** Diederick Penning, Jasper Tausendfreund, M. Azad Naryapragi, Kostan W. Reisinger, Pieter Joosse, Erik Tanis, Tim Schepers

**Affiliations:** Trauma Unit, Department of Surgery, Amsterdam UMC, University of Amsterdam, Amsterdam, The Netherlands; Department of Trauma Surgery, Noordwest Ziekenhuisgroep, Alkmaar, The Netherlands; Trauma Unit, Department of Surgery, Amsterdam UMC, University of Amsterdam, Amsterdam, The Netherlands; Trauma Unit, Department of Surgery, Amsterdam UMC location VUmc, Amsterdam, The Netherlands; Department of Trauma Surgery, Noordwest Ziekenhuisgroep, Alkmaar, The Netherlands; Department of Trauma Surgery, Noordwest Ziekenhuisgroep, Alkmaar, The Netherlands; Trauma Unit, Department of Surgery, Amsterdam UMC, University of Amsterdam, Amsterdam, The Netherlands

**Keywords:** ankle fracture, ankle dislocation, external fixation, splint, temporary management

## Abstract

Ankle fracture-dislocations may require delayed internal fixation. Our aim was to compare acute open reduction and internal fixation (ORIF) with delayed ORIF, using external fixation or cast splint in ankle fracture-dislocations. Factors that affect the rates of re-operation and Surgical site infection (SSI) were identified. In this retrospective cohort study, patients were included with open and closed ankle fracture-dislocations treated with ORIF from two large peripheral hospitals and one academic center in the Netherlands. This study included 447 patients with an ankle fracture-dislocation. In the multivariate analysis, the difference between surgery <48 hours compared to bridging with cast or external fixation had no significant influence on unscheduled re-operation or SSI. Higher body mass index (BMI) and open fractures had a significant positive correlation with re-operation while diabetes mellitus (DM) and open fractures correlated with SSI. In patients with open fractures, there was also no significant difference in outcome between acute or delayed internal fixation. We suggest that it is safe to perform primary ORIF on all dislocated ankle fractures if the soft tissue injury allows surgery within 48 hours. When significant swelling is present, patients with well-reduced fractures and with no soft tissue injury could be treated safely with a cast until delayed ORIF is possible.

**Level of Evidence**: *Therapeutic level 2B (retrospective cohort study)*

“Unstable ankle fractures with indication for surgical reduction are primarily treated with open reduction and internal fixation (ORIF).”

## Introduction

Ankle fractures are among the most frequently observed injuries in emergency departments, with an incidence of 187 per 100,000 in the adult population.^1,2^ The demographics of patients with ankle fractures indicate an increased occurrence in the adolescent age group, and the primary mechanisms of injury include falls and sports-related incidents.^3,4^ Notably, there is an observed rise in the incidence of ankle fractures, possibly due to a more active elderly population. In addition, the highest age-specific incidence in women occurs in the decade between 75 and 84 years of age.^5-8^

Ankle fractures can vary in type and severity, as depicted on the Lauge-Hansen classification (LH), Weber classification, or the number of fractured malleoli, often referred to as Pott’s classification.^9-11^

Unstable ankle fractures with indication for surgical reduction are primarily treated with open reduction and internal fixation (ORIF).^12^ However, ankle fractures with soft tissue damage, including significant swelling, may require delayed internal fixation.^13,14^ Therefore, a bridging cast or external fixation can be used to temporary stabilize the fracture until definitive surgery.^15-19^ External fixation offers superior fracture stabilization and easier soft tissue monitoring compared to a bridging cast.^18,19^ However, patients treated with external fixation typically experience a longer hospital admission time, and the treatment may pose psychological challenges for them.^20,21^ Additionally, the higher costs associated with prolonged hospital stay following external fixation result in a more expensive overall treatment.^22^

Surgical site infections (SSIs) are among the most common and significant complications following ankle fracture surgery.^23-25^ Various risk factors are associated with the prediction of SSIs.^26^ Open fractures, generally classified using the Gustilo classification, are associated with an increased rate of SSI.^27^ Additionally, the use of tobacco, diabetes mellitus (DM), a higher classification of the American Society of Anesthesiologists’ criteria (ASA), and an elevated body mass index (BMI) hold significant predictive value for the development of SSIs in ankle fractures.^26,28,29^ Dislocation fractures of the tibio-talar joint result from high-energy trauma with concomitant soft tissue damage.^30^ When this soft tissue damage is severe, closure of the wound following ORIF may not be possible. Consequently, these fractures may require delayed internal fixation.^31-34^

The aim of this study was to compare the use of acute ORIF with delayed ORIF, following the use of external fixation or cast splint in ankle fracture-dislocations, in relation to the risk of re-operation and postoperative SSI. Additionally, we compare the rates of re-operation and SSI, as well as the associated risk factors in a subgroup of open ankle fractures.

## Materials and Methods

### Study Design

This retrospective cohort study included all consecutive patients with an ankle fracture-dislocation who were surgically treated between January 2015 and December 2020. Patients were identified from two large peripheral hospitals and one academic center in the Netherlands, using the electronic patient file (EPF) using procedural and diagnostic codes. Data were extracted by two authors (DP and JT) and a consensus meeting was scheduled in case of any discrepancies (with ET, PJ, and TS).

**Figure 1.**
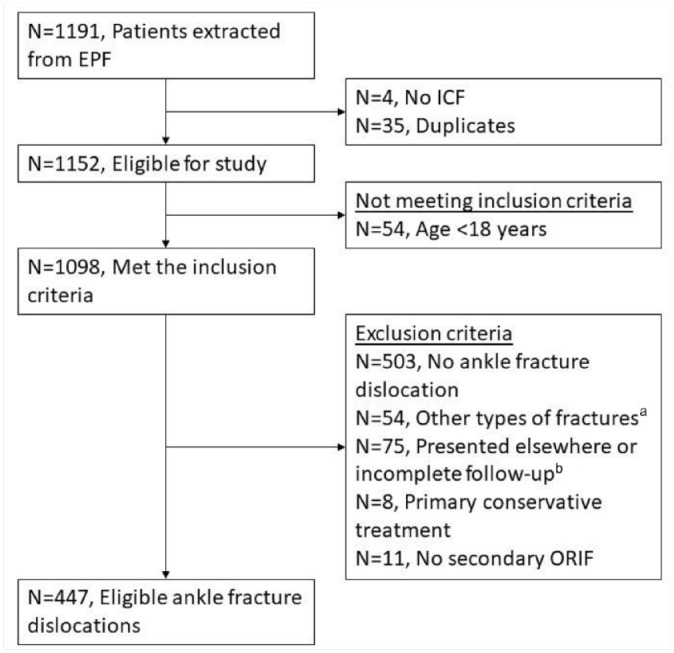
Inclusion flowchart. ^a^Calcaneus, distal tibia, cruris, metatarsal, talus. ^b^Missing follow-up visits or follow-up radiology. EPF: electronic patient file, ICF: informed consent form, ORIF: open reduction and internal fixation.

Figure 1 shows the inclusion flowchart.

Inclusion criteria: age ≥ 18 years and/or skeletally mature, surgical treatment of an ankle fracture-dislocation with ORIF, minimal follow-up of 12 months.

Exclusion criteria: other type of fracture than ankle fracture, less than 50% talar dislocation, follow-up less than 12 months, started with non-surgical treatment, external fixation without secondary ORIF.

Ankle fractures were classified as a dislocated ankle fractures when there was a minimum of 50% displacement of the talus compared to the tibial pilon joint surface, in either the anteroposterior (AP) view, or the lateral view of the first radiographic image following trauma.^30^ In addition, patients in which a documented closed reduction was performed prior to the initial radiographic imaging were included.

Patient demographic characteristics included gender, age, weight, BMI, DM, smoking status, alcohol consumption (more than two units/day), and ASA classification.

Injury-related variables included date of injury, fracture type classified using Weber, Lauge-Hansen, and the number of malleoli involved.^9-11^ The presence of a fracture of the posterior malleolus was also registered separately. Furthermore, open fractures were classified according to the Gustilo classification. The subgroup of patients with open dislocated ankle fractures will be analyzed separately.

Treatment-related variables included date of surgery, time from injury to ORIF (less than 48 hours, more than 48 hours stabilized with cast, more than 48 hours stabilized with external fixation), reduction, use of cast, time from injury to external fixation, time from external fixation to ORIF. The applied cast was usually a circular split cast or back slab to provide room for swelling.

Outcome included SSI, type of infection (superficial or deep), re-operation, and need for (partial) removal of the implant. Postoperative infections were diagnosed and classified as deep or superficial by the clinician based on the CDC criteria.^35^ Other registered complications were postoperative bleeding and skin necrosis.

This study was judged by the local medical ethics testing committee (METC AMC) as a non-WMO study. Patients from the academic center were asked to sign no-objection forms according to the METC and patients from the two peripheral hospitals were not asked for a declaration of no-objection, according to the judgment of their local METC.

### Statistical Analysis

Statistical analyses were performed using IBM Statistical Package for Social Sciences (SPSS) 26.0.0.1.

For statistical data analysis, Pearson's chi-square and Fisher’s exact tests were used to compare the categorical variables. Continuous variables were tested for normality with the Kolmogorov–Smirnov and Shapiro–Wilk tests. Non-normally divided variables were compared using the Mann–Whitney *U* test and displayed as median with interquartile range (IQR). Normally distributed variables were compared using a *t*-test and displayed with a mean and standard deviation.

Binary logistic regression was used for univariate and multivariate analyses to calculate if the outcome variables were significantly dependent on demographic, injury-related or treatment-related variables. Odds ratios (OR) with a 95% confidence interval (CI) were also calculated using binary logistic regression.

For all statistical tests, a *P*-value of <.05 was considered statistically significant.

## Results

We included 447 patients with an ankle fracture-dislocation in our study. Table 1 shows the patient characteristics of the total group and the subgroups of primary fixation within 48 hours following trauma, secondary fixation stabilized with cast, or stabilized with external fixation. Significant differences between the groups were age, ASA score, percentage of patients in which pre-operative reduction has been performed, amount of malleoli involved, open fractures, and re-operation rate. Patients treated with temporary casts were significantly younger and had lower ASA classification. Patients treated with external fixations had a lower frequency of reduction prior to surgery and a higher rate of trimalleolar fracture. The open fractures were more frequently treated with external fixation. Patients treated with external fixation had a higher rate of re-operation.

**Table 1.** Baseline Characteristics of all Patients With Ankle Fracture-Dislocations.

	Primary ORIF <48 hours, n = 162	Secondary ORIF, Cast, n = 226	Secondary ORIF, external fixation, n = 59	Total, n = 447	*P*-value
Male gender (male, %)	60 (37.0%)	92 (40.7%)	27 (45.8%)	179 (40.0%)	ns
Age, median, in years [IQR]	58.5 [25]	50 [32]	59 [31]	54 [30]	**<.01**
BMI, median [IQR]	27 [5]	27 [6]	28 [8]	27 [6]	ns
ASA score, n (%)	162 (100%)	226 (100%)	59 (100%)	447 (100%)	**.01**
ASA-1 ASA-2 ASA-3 ASA-4	48 (29.6%) 88 (54.3%) 23 (14.2%) 3 (1.9%)	89 (39.4%) 108 (47.8%) 29 (12.8%) 0 (0.0%)	11 (18.6%) 34 (57.6%) 12 (20.3%) 2 (3.4%)	148 (33.1%) 230 (51.5%) 64 (14.3%) 5 (1.1%)	
Diabetes mellitus, n (%)	15 (9.3%)	17 (7.5%)	4 (6.9%)	36 (8.1%)	ns
Alcohol^a^, *n (%)*	18 (15.5%)	27 (15.6%)	7 (23.3%)	52 (16.3%)	ns
Smoking, n (%)	32 (26.9%)	39 (22.2%)	10 (30.3%)	81 (24.7%)	ns
Reduction performed, n (%)	149 (92.0%)	214 (95.1%)	47 (79.7%)	410 (91.9%)	**<.01**
Type of fracture, n (%)	162 (100%)	226 (100%)	59 (100%)	447 (100%)	**<.01**
Unimalleolar Bimalleolar Trimalleolar	17 (10.5%) 57 (35.2%) 88 (54.3%)	33 (14.6%) 57 (25.2%) 136 (60.2%)	1 (1.7%) 13 (22.0%) 45 (76.3%)	51 (11.4%) 127 (28.4%) 269 (60.2%)	
Posterior malleolar fracture, n (%)	107 (66.0%)	153 (67.7%)	47 (79.7%)	307 (68.7%)	ns
Weber classification, n (%)	162 (100%)	226 (100%)	59 (100%)	447 (100%)	ns
Not possible Weber A Weber B Weber C	3 (1.9%) 2 (1.2%) 98 (60.5%) 59 (36.4%)	3 (1.3%) 4 (1.8%) 149 (65.9%) 70 (31.0%)	1 (1.7%) 0 (0.0%) 40 (67.8%) 18 (30.5%)	7 (1.6%) 6 (1.3%) 287 (64.2%) 147 (32.9%)	
Lauge-Hansen classification n (%)	159 (100%)	226 (100%)	57 (100%)	442 (100%)	ns
Not possible Supination-adduction Pronation-abduction Supination-external rotation Pronation-external rotation	7 (4.4%) 1 (0.6%) 2 (1.3%) 96 (60.4%) 53 (33.3%)	8 (3.5%) 3 (1.3%) 1 (0.4%) 149 (65.9%) 65 (28.8%)	1 (1.8%) 0 (0.0%) 0 (0.0%) 39 (68.4%) 17 (29.8%)	16 (3.6%) 4 (0.4%) 3 (0.7%) 284 (64.3%) 135 (30.5%)	
Open fracture, n (%)	48 (29.6%)	10 (4.4%)	22 (37.3%)	80 (17.9%)	**<.01**
Gustilo classification, n (%)	48 (100%)	10 (100%)	22 (100%)	80 (100%)	**<.01**
Gustilo I Gustilo II Gustilo III	19 (39.6%) 15 (31.3%) 14 (29.2%)	8 (80.0%) 2 (20.0%) 0 (0.0%)	4 (18.2%) 13 (59.1%) 5 (22.7%)	31 (38.8%) 30 (37.5%) 19 (23.8%)	
Time onset to ORIF, median, in days [IQR]	0 [0-1]	10 [8-13]	12 [9-14]		
Time Fix-ex to ORIF, median, in days [IQR]	N.A.	N.A.	10 [8-13]		
Re-operation n (%)	19 (11.7 %)	22 (9.7%)	12 (20.3%)	53 (11.9%)	**.04**
Indication for re-operation					
Malunion Secondary dislocation Arthrodesis Other Infection Vascular damage Split skin graft	4 (2.6%) 6 (3.7%) 1 (0.6%) 2 (1.2%) 5 (3.1%) 0 (0.0%) 1 (0.6%)	2 (0.8%) 8 (3.5%) 0 (0.0%) 4 (1.8%) 8 (3.5%) 0 (0.0%) 0 (0.0%)	0 (0.0%) 2 (3.4%) 4 (6.8%) 2 (3.4%) 3 (5.1%) 1 (1.7%) 0 (0.0%)	6 (1.3%) 16 (3.6%) 5 (1.1%) 8 (1.8%) 16 (3.6%) 1 (0.2%) 1 (0.2%)	
Surgical site infection (SSI), n (%)	17 (10.5%)	25 (11.1%)	8 (13.6%)	50 (11.2%)	ns
Type of SSI, n (%)	17 (10.5%)	25 (11.1%)	8 (13.6%)	50 (11.2%)	ns
Superficial Deep	11 (6.8%) 6 (3.7%)	13 (5.80%) 12 (5.3%)	5 (8.5%) 3 (5.1%)	28 (6.3%) 21 (4.7%)	
Implant removal, n (%)	52 (32.1%)	94 (41.6%)	15 (25.4%)	161 (36.0%)	ns
Plate Syndesmotic screw only	48 (29.6%) 4 (2.5%)	83 (36.7%) 11 (4.9%)	12 (20.3%) 3 (5.1%)	143 (32.0%) 18 (4.0%)	

a Alcohol more than 2 units/day.

All statistically significant values <0.05 are presented in bold.

Table 2 shows the correlation between each variable and re-operation. This was done using univariate and multiple logistic regression analysis. Therefore, the OR with concomitant p-value is provided for every variable. Positive correlating variables in the univariate analysis were BMI, BMI with a cut-off of 30, external fixation, and open fracture. In the multivariate analysis, BMI and open fractures had a positive correlation with re-operation, indicating that the risk of re-operation increases with higher BMI and in patients with open fractures.

**Table 2.** Logistic Regression for Risk at Re-operation in Ankle Fracture-Dislocations (n = 53).

Variable	OR (95% CI)	*P*-value
***Univariate***		
**Age**	1.00 (0.98-1.01)	.70
**Alcohol**	0.61 (0.21-1.81)	.38
**ASA 1/2 or 3/4**	1.14 (0.53-2.45)	.74
**BMI**	1.09 (1.03-1.16)	**<.01**
**BMI <30 or >30**	2.48 (1.30-4.73)	**.01**
**Diabetes mellitus**	1.55 (0.61-3.91)	.36
**Fix-ex**	2.16 (1.06-4.40)	**.03**
**Gender**	1.22 (0.67-2.22)	.51
**Lauge-Hansen** Not possible Supination-adduction Pronation-abduction Supination-external rotation Pronation-external rotation	Reference 0.00 0.80 (0.17-3.68) 1.29 (0.27-6.09) 3.5 (0.21-58.78)	.15 .77 .75 .38
**Open fracture**	3.73 (2.01-6.90)	**<.01**
**Pre-operative re-dislocation**	0.81 (0.18-3.60)	.78
**Primaire ORIF/Sec. ORIF/Fix Ex** <48 hr Sec. ORIF External fixation	Reference 0.81 (0.42-1.56) 1.92 (0.87-4.25)	.53 .11
**Smoking**	1.34 (0.63-2.84)	.45
**Tertius**	0.60 (0.34-1.08)	.09
**UniBiTrimalleolar** Unimalleolar Bimalleolar Trimalleolar	Reference 0.83 (0.33-2.07) 0.62 (0.27-1.46)	.22 .69 .28
**Weber** **Not possible** **A** **B** **C**		.13
***Multivariate***		
**BMI** **Open** **Fix-ex**	1.08 (1.02-1.15) 4.52 (2.24-9.13) 1.22 (0.51-2.94)	**.02** **<.01** .65
**BMI** **Open**	1.08 (1.12-1.15) 4.68 (2.35-9.30)	**.01** **<.01**
**BMI >30** **Open** **Fix-ex**	2.20 (1.12-4.31) 4.52 (2.25-9.12) 1.23 (0.52-2.95)	**.02** **<.01** .64
**BMI >30** **Open**	2.22 (1.14-4.34) 4.69 (2.36-9.30)	**.02** **<.01**

All statistically significant values <0.05 are presented in bold.

External fixation, however, had no correlation with re-operation, and BMI with a cut-off of 30 had a stronger correlation than BMI as a continuous variable.

Table 3 shows the outcome of the univariate and multiple logistic regressions to compare the influence of each variable on SSI.

**Table 3.** Logistic Regression for Risk at SSI in Ankle Fracture-Dislocations (n = 50).

Variable	OR (95% CI)	*P*-value
***Univariate***		
**Age**	1.02 (1.00-1.03)	.08
**Alcohol**	0.84 (0.31-2.27)	.73
**ASA 1/2 or 3/4**	1.23 (0.57-2.66)	.60
**BMI**	1.05 (0.98-1.11)	.17
**BMI <30 or >30**	1.27 (0.62-2.60)	.52
**Diabetes mellitus**	2.50 (1.07-5.83)	**.03**
**Fix-ex**	1.29 (0.57-2.90)	.54
**Gender**	1.00 (0.55-1.82)	.99
**Smoking**	0.91 (0.39-2.08)	.82
**Lauge-Hansen** Not possible Supination-adduction Pronation-abduction Supination-external rotation Pronation-external rotation	Reference 0.0 (0.00) 0.86 (0.19-3.97) 0.94 (0.20-4.53) 3.50 (0.21-58.77)	.63 1.00 .85 .94 .38
**Open fracture**	2.18 (1.13-4.23)	**.02**
**Pre-operative re-dislocation**	0.88 (0.20-3.93)	.86
**Primary ORIF/Sec. ORIF/Fix Ex** <48 hours Sec. ORIF External fixation	Reference 1.05 (0.55-2.02) 1.33 (0.54-3.27)	.58 .88 .54
**Posterior malleolar fracture**	1.08 (0.57-2.04)	.82
**Uni-, Bi-, and Trimalleolar** Unimalleolar Bimalleolar Trimalleolar	Reference 0.97 (0.32-2.90) 1.29 (0.48-3.47)	.44 .95 .62
**Weber** **Not possible** **A** **B** **C**		.64
***Multivariate***		
**DM** **Open**	2.49 (1.06-5.87) 2.21 (1.14-4.31)	.**04** **.02**

All statistically significant values <0.05 are presented in bold.

Positively correlating variables in the univariate analysis were DM and open fractures. In the multivariate analysis, DM and open fractures led to an increased risk of SSI.

A subgroup analysis for patients with open ankle fractures was performed. This group is shown in Table 4. There was a significant difference in Gustilo's classification between the methods of treatment. A univariate and multiple regression analysis was performed to study the influence of every variable on outcome. The results for re-operation are shown in Table 5, and for SSI in Table 6.

**Table 4.** Characteristics of Patients With Open Ankle Fractures.

*Open ankle fractures* *Patients, n*	Primary ORIF N = 48	Secondary ORIF Cast N = 10	Secondary ORIF Fix-ex N = 22	Total N = 80	*P*-value
Male gender (Male, %)	22 (45.8%)	6 (60.0%)	13 (59.1%)	41 (51.2%)	ns.
Age, median, in years [IQR]	61.5 [37]	46.5 [32]	53.5 [32]	60.5 [36]	ns.
BMI, median [IQR]	27.4 [7]	31.0 [13]	26.9 [8]	27.6 [8]	ns.
BMI >30, n (%)	11 (31.4%)	4 (40.0%)	5 (22.7%)	20 (34.5%)	ns.
ASA score, n (%)					
ASA-1 ASA-2 ASA-3 ASA-4	11 (22.9%) 27 (56.3%) 8 (16.7%) 2 (4.2%)	3 (30.0%) 6 (60.0%) 1 (10.0%) 0	3 (13.6%) 14 (63.6%) 4 (18.2%) 1 (4.5%)	17 (21.3%) 47 (58.8%) 13 (16.3%) 3 (3.8%)	ns.
Diabetes mellitus, n (%)	3 (6.3%)	2 (20.0%)	2 (9.1%)	7 (8.9%)	ns.
Alcohol^a^, n (%)	1 (2.1%)	0	2 (9.1%)	3 (6.1%)	ns.
Smoking, n (%)	9 (18.8%)	0	4 (18.2%)	13 (16.3%)	ns.
Type of fracture, n (%)					
Unimalleolar Bimalleolar Trimalleolar	8 (16.7%) 22 (45.8%) 18 (37.5%)	1 (10.0%) 7 (70.0%) 2 (20.0%)	1 (4.5%) 7 (31.8%) 14 (63.6%)	10 (12.5%) 36 (45.0%) 34 (42.5%)	ns.
Posterior malleolar fracture, n (%)	24 (50.0%)	2 (20.0%)	14 (63.6%)	40 (50.0%)	ns.
Lauge-Hansen classification, n (%)					ns.
Not possible Supination-adduction Pronation-abduction Supination-external rotation Pronation-external rotation	5 (10.4%) 1 (2.1%) 1 (2.1%) 24 (51.1%) 16 (33.3%)	0 2 (20.0%) 0 4 (40.0%) 4 (40.0%)	0 0 0 14 (66.7%) 7 (33.3%)	5 (6.4%) 3 (3.8%) 1 (1.3%) 42 (53.8%) 27 (34.6%)	
Gustilo classification, n (%)					**.01**
Gustilo I Gustilo II Gustilo III	19 (39.6%) 15 (31.3%) 14 (29.2%)	8 (80.0%) 2 (20.0%) 0	4 (18.2%) 13 (59.1%) 5 (22.7%)	31 (38.8%) 30 (37.5%) 19 (23.8%)	
Time onset to ORIF (in days)		12.5 [7]	12.0 [7]		ns.
Time Fix-ex to ORIF			11.0 [7]		
Re-operation, n (%)	12 (25.0%)	3 (30.0%)	6 (27.3%)	21 (26.3%)	ns.
Malunion Secondary dislocation Arthrodesis Other Infection Vascular damage Pseudo-arthritis Split skin graft	3 (6.3%) 3 (6.3%) 0 1 (2.1%) 4 (8.33%) 0 0 1 (2.1%)	0 1 (10%) 0 0 2 (20%) 0 0 0	0 0 4 (18.2%) 1 (4.5%) 1 (4.5%) 0 0 0	3 (3.8%) 4 (5.0%) 4 (5.0%) 2 (2.5%) 7 (8.9%) 0 0 1 (1.3%)	
Surgical site infection (SSI), n (%)	9 (18.8%)	3 (30.0%)	3 (13.6%)	15 (18.8%)	ns.
Superficial Deep	3 (6.3%) 5 (10.4%)	0 3 (30.0%)	2 (9.1%) 1 (4.5%)	5 (6.4%) 9 (11.3%)	
Implant removal, n (%)					
Plate Syndesmotic screw only	14 (29.2%) 2 (4.2%)	5 (50.0%) 0	3 (13.6%) 2 (9.1%)	22 (27.5%) 4 (5.0%)	ns.

a Alcohol more than 2 units/day.

All statistically significant values <0.05 are presented in bold.

**Table 5.** Logistic Regression for the Risk at Re-operation in Open Fractures (n = 21).

Variable	OR (95% CI)	*P*-value
***Univariate***		
**Age**	1.01 (0.98-1.03)	.84
**Alcohol**	0.00	1.00
**ASA 1/2 or 3/4**	1.36 (0.41-4.52)	.61
**BMI**	1.13 (1.01-1.25)	**.03**
**BMI <30 or >30**	2.29 (0.73-7.16)	.15
**Diabetes mellitus**	8.75 (1.55-49.42)	**.01**
**Fix-ex**	1.08 (0.36-3.25)	.90
**Gender**	1.58 (0.58-4.32)	.37
**Lauge-Hansen** Not possible Supination-adduction Pronation-abduction Supination-external rotation Pronation-external rotation	Reference 0.00 1.09 (0.11-11.01) 2.35 (0.23-24.10)	1.00 1.00 .94 .47
**Pre-operative re-dislocation**	0.00	1.00
**Primaire ORIF/Sec. ORIF/Fix Ex** <48h Sec. ORIF External fixation	Reference 1.29 (0.29-5.78) 1.13 (0.36-3.53)	.74 .84
**Smoking**	0.31 (0.06-1.61)	.17
**Posterior malleolar fracture**	0.52 (0.19-1.44)	.21
**Uni-, Bi-, Tri-malleolar** Unimalleolar Bimalleolar Trimalleolar	Reference 0.58 (0.13-2.49) 0.39 (0.09-1.77)	.46 .22
***Multivariate***		
**BMI** **DM**	1.06 (0.93-1.21) 7.62 (0.61-95.70)	.36 .12

All statistically significant values <0.05 are presented in bold.

**Table 6.** Logistic Regression for the Risk at SSI in Open Fractures (n = 15).

Variable	OR (95% CI)	*P*-value
***Univariate***		
**Age**	1.01 (0.98-1.04)	.61
**Alcohol**	0.00	1.00
**ASA 1/2 or 3/4**	1.61 (0.44-5.92)	.48
**BMI**	1.14 (1.02-1.27)	**.03**
**BMI <30 or >30**	2.95 (0.88-9.94)	.08
**Diabetes mellitus**	7.39 (1.45-37.69)	**.02**
**Fix-ex**	0.61 (0.15-2.39)	.47
**Gender**	1.25 (0.41-3.86)	.69
**Lauge-Hansen** Not possible Supination-adduction Pronation-abduction Supination-external rotation Pronation-external rotation	Reference 0.00 0.00 0.94 (0.09-9.60) 0.91 (0.08-9.99)	1.00 1.00 .96 .94
**Pre-operative re-dislocation**	0.00	1.00
**Primaire ORIF/Sec. ORIF/Fix Ex** <48h Sec. ORIF External fixation	Reference 1.86 (0.40-8.62) 0.68 (0.17-2.82)	.43 .60
**Smoking**	0.59 (0.11-3.15)	.53
**Posterior malleolar fracture**	0.61 (0.19-1.90)	.39
**Uni-, Bi-, Tri-malleolar** Unimalleolar Bimalleolar Trimalleolar	Reference 0.56 (0.12-2.75) 0.40 (0.78-2.10)	.48 .28
***Multivariate***		
**BMI** **DM**	1.09 (0.95-1.25) 3.31 (0.37-29.98)	.22 .29

All statistically significant values <0.05 are presented in bold.

In this group, higher BMI and DM had a correlation with both re-operation and SSI in the univariate analysis but were not significant in the multivariate analysis. Also, in this group, external fixation had no correlation with both re-operation and SSI.

## Discussion

In this cohort, there was no significant increase in re-operation and SSI when external fixation was used before ORIF in patients with ankle fracture-dislocations. Even though external fixation is particularly reserved for ankle fractures with more severe soft tissue damage. BMI and open fractures were related to a higher frequency of re-operation. DM and open fractures were related to a higher frequency of SSI. Open fractures and DM have also been identified as risk factors in ankle fractures without dislocation.^36,37^ Specifically for patients with DM, non-surgical treatment of ankle fractures is associated with high complication rates, although a different study did not find any correlation between surgical timing and postoperative complications.^38,39^ However, studies about risk factors in patients with ankle fracture-dislocations are limited.

Open fractures, characterized by the highest degree of soft tissue damage, were associated with increased rates of re-operation and SSI in our cohort. Consequently, we focused on this subgroup to identify additional risk factors. Among patients with open ankle fracture-dislocations, no correlation was found with any of the variables, including the strategy of treatment (direct or delayed). In a previous multivariate analysis, significant predictors of infection in open ankle fractures were identified: male gender, DM, smoking, use of immunosuppressants, lateral wound localization, and time to closure.^40^

There were no indications that the use of external fixation reduced the risk of SSI or re-operation. Therefore, we suggest that all efforts should be made to surgically treat patients with ankle fracture-dislocations within 48 hours following trauma. This is supported by previous studies on the timing of surgery for unstable ankle fractures, where early surgery decreases the risk of complications.^41^ Additionally, a study by Tanoglu compared 1-stage and 2-stage surgery in ankle fracture-dislocations and concluded that 2-stage surgery is a safe option, but functional outcome scores were not superior following external fixation.^42^ In some cases, for example, when swelling prevents complete closure following internal fixation, the window for surgery within 48 hours is not feasible, and the choice of treatment is limited to delayed ORIF using either external fixation or cast. Patients with open ankle fracture-dislocations and a higher risk for re-operation or infection could be treated using an external fixator because this offers an easier view of the wound and more stabilization compared to a cast. Our data do not show a higher percentage of dislocation in a cast or increased risk for re-operation following trimalleolar fractures. This is in contrast to Buyukkuscu et al,^18^ and Wawrose et al^19^ who have found external fixation reduces the risk of loss of reduction and skin necrosis, compared to bridging with the cast. These studies respectively consisted of 56 and 117 patients and were therefore considerably smaller than our cohort.

Both Wawrose and Buyukkuscu found higher percentages of re-dislocation in a cast, compared to our cohort with 3.5% re-dislocation (Wawrose 50.0% and Buyukkuscu 24.6%). Because our cohort is retrospective, it is possible that more stable fractures were selected for bridging with a cast. This might be the reason for the comparable re-dislocation rates between the groups. However, the two studies of Wawrose and Buyukkuscu were also of retrospective nature and therefore, the design of our study would not be completely responsible for the difference in re-dislocation. It does remain important to recognize the patients with fractures that are more likely to re-dislocate than others. The risk factors for re-operation suggest we should be cautious in selecting patients for temporary stabilization with a cast. These risk factors include fractures with larger posterior malleolar fragments, even though this was not supported by our results.^30,43^ Ankle fracture-dislocations are also associated with more long-term symptoms, including pain, loss of ROM, and increased problems with ADL and sport.^44^ However, the number of studies comparing the functional outcome of different treatment strategies in patients with ankle fracture-dislocation is limited.^18,42^ Long-term follow-up of a larger-sized prospective cohort including functional outcomes of patients with ankle fracture-dislocations would provide us with a better comprehension of cartilage damage, which has been studied in ankle fractures without dislocation.^45^ Mid-term and long-term functional outcomes have also been studied in relatively small cohorts.^46,47^ Therefore, these subjects might be the scope of future studies.

A limitation of this study is the retrospective character, which includes the risk of selection bias. Clinicians made the decision about the choice of treatment. This may explain the differences in age, ASA class, and type of injury between the groups. This was because the treating clinicians could have made the decision to treat patients with more severe fractures, frail patients, or severely damaged soft tissue injuries differently than others. We aimed to decrease the influence of selection bias by performing the multivariate regression analysis and therefore identify independent risk factors.

In our cohort, patients with higher BMI and DM had an increased risk for re-operation or SSI.

For patients with closed ankle fracture-dislocations without these risk factors, the clinician could consider bridging time to surgery with cast when fixation within 48 hours is not possible. This is because we did not find that the use of external fixation significantly decreases the chance of SSI or re-operation.

Based on this large retrospective cohort study, we suggest that it is safe to perform primary ORIF on all dislocated ankle fractures if the soft tissue injury allows surgery within 48 hours. When significant swelling is present, temporary immobilization is a safe option to allow for surgery when swelling is reduced. Patients with well-reduced fractures and with no soft tissue injury could be treated safely with a cast until delayed ORIF is possible. Patients with higher BMI and DM have an increased risk for re-operation or SSI. This study guides clinicians to select patients for each of the treatment options.
